# Primary amyloidosis of conjunctiva

**DOI:** 10.1002/jha2.567

**Published:** 2022-09-09

**Authors:** Karuna Jha, Maitreyee Bhattacharyya

**Affiliations:** ^1^ Clinical Hematology Institute of Hematology and Transfusion Medicine Medical College Kolkata India; ^2^ Institute of Hematology and Transfusion Medicine Medical College Kolkata India

1

A 64‐year old male presented in ophthalmology OPD for follow up for refractive errors. He had been wearing bifocal lenses from last 1 year. On local examination, medial canthus of left eye showed a pale yellowish plaque. The rest of the ocular examination and contralateral eye were normal. Slit lamp examination showed a confluent fusiform papule (Figure 1A,B). Computed tomography (CT) scan of the orbit showed no abnormality. Surgical excision was done and tissue sent for histopathological examination showed deposition of a pink homogeneous material in the fibrocollagenous tissue beneath the conjunctival epithelium (Figure 1,C). Congo red stain followed by polarizing microscopy confirmed the pink material to be amyloid with apple green birefringence (Figure 1D,E). Immunohistochemistry (IHC) showed lambda light chain restriction. A diagnosis of conjunctival amyloidosis was made. The patient was then evaluated to rule out systemic amyloidosis or plasma cell dyscrasia. The patient was asymptomatic with no history of trauma, systemic illness, and family history was non‐contributory. General and systemic examinations were unremarkable. Complete hemogram, urea, creatinine, liver function test, erythrocyte sedimentation rate, blood glucose, and serum calcium were within normal limits. No abnormality was detected on skeletal survey, electrocardiography, 2D‐echocardiography, and ultrasonography of abdomen. Serum protein electrophoresis, serum immunofixation electrophoresis, serum free light chain assay, 24‐h urine protein, and urine protein electrophoresis were non‐contributory. Bone marrow showed mildly increased plasma cells without light chain restriction on immunohistochemistry. Finally, a diagnosis of primary conjunctival amyloidosis was made and the patient was asked to follow up.

**FIGURE 1:**
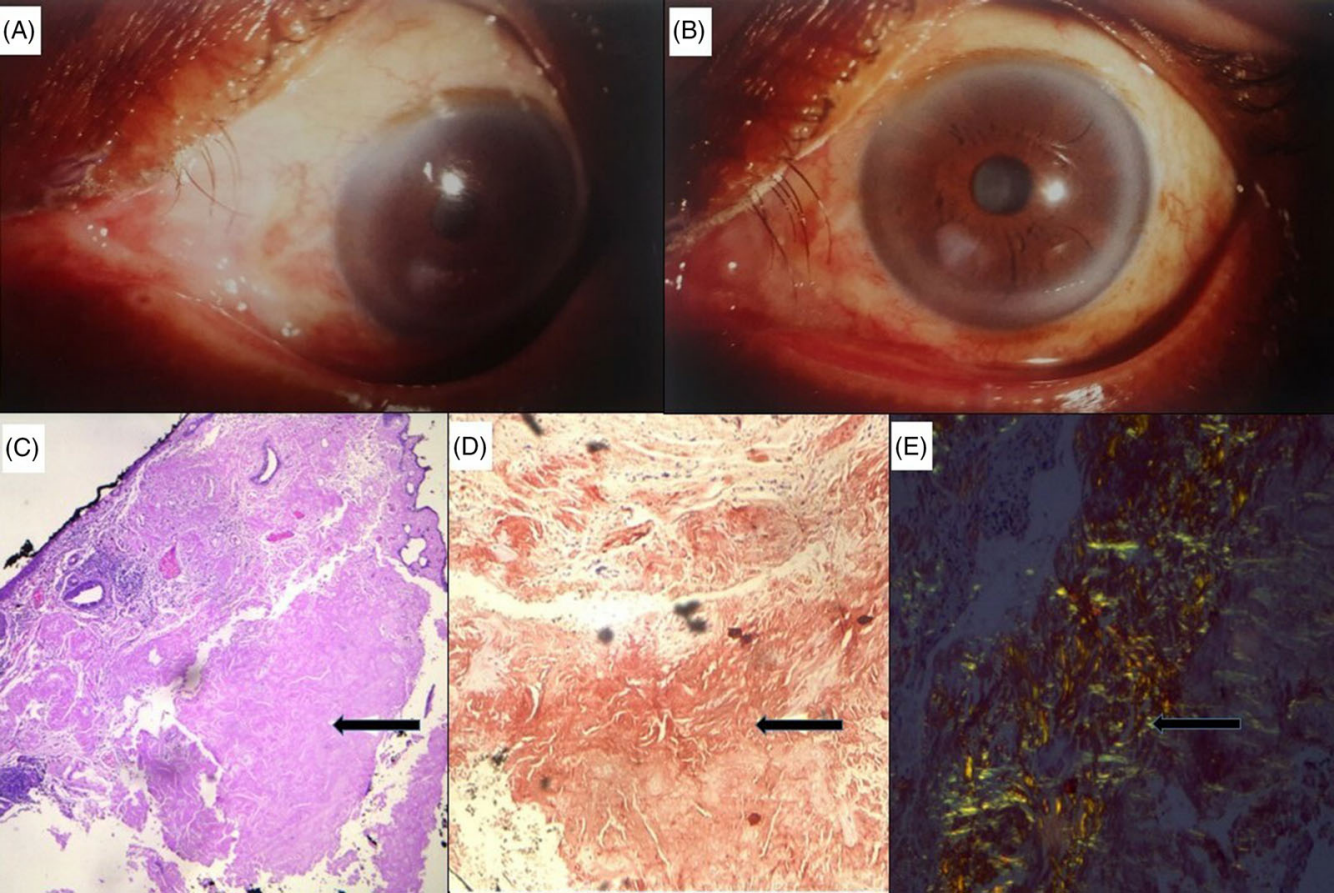
(A and B) Slit lamp examination of left eye showing pale yellowish plaque in the median canthus. (C) Hematoxylin and eosin stain showing deposition of pink homogeneous material beneath conjunctical epithelium (magnification 5×). (D) Congo red stain on pink homogeneous material. (E) Polarizing microscopy showing apple green birefringence

Conjunctival amyloidosis occurs secondary to local inflammation and primary form of the disease is very rare. In a previous series published on ocular amyloidosis, only two of 26 ocular cases collected over 30 years had conjunctival involvement. Identification of the disease is a daunting task owing to protean manifestations. Differentiating it from conjunctival inflammation and malignancies is essential. Evaluation for systemic causes is important as the treatment modalities are completely different. While localized form might just need observation or surgical debulking, systemic AL amyloidosis or myeloma would necessitate administration of specific chemotherapy. A long‐term follow up is also necessary as recurrence is a known phenomenon and evolution into systemic disease is also a possibility.

## AUTHOR CONTRIBUTIONS

*Concepts design, definition of intellectual content, Literature search, data analysis, manuscript preparation, and manuscript editing*: Karuna Jha. *Concepts design, definition of intellectual content, manuscript editing*: Maitreyee Bhattacharyya.

## CONFLICT OF INTEREST

The authors declare they have no conflicts of interest.

## FUNDING INFORMATION

The authors received no specific funding for this work.

2

## ETHICS STATEMENT

No research on human was performed on this study.

## PATIENT CONSENT STATEMENT

Informed consent was taken from the patient.

## Data Availability

The data that support the findings of this study are available from the corresponding author upon reasonable request.

